# Sustaining and strengthening community resilience throughout the COVID-19 pandemic and beyond

**DOI:** 10.1177/1757913920949582

**Published:** 2020-08-21

**Authors:** J South, J Stansfield, R Amlôt, D Weston

**Affiliations:** Health Improvement Directorate, Public Health England, UK; Health Improvement Directorate, Public Health England, UK; School of Health & Community Studies, Leeds Beckett University, UK; Behavioural Science Team, Emergency Response Department, Health Protection, Public Health England, UK; Behavioural Science Team, Emergency Response Department, Health Protection, Public Health England, UK

*Jane South writes about the community response to the Covid-19 emergency in the UK and how public health has a role in strengthening community resilience through the recovery. This article reflects on the huge community response as part of the pandemic and draws on PHE and WHO Europe guidance on communities to discuss how this could be applied in public health practice as we move into recovery.*

**Figure:**
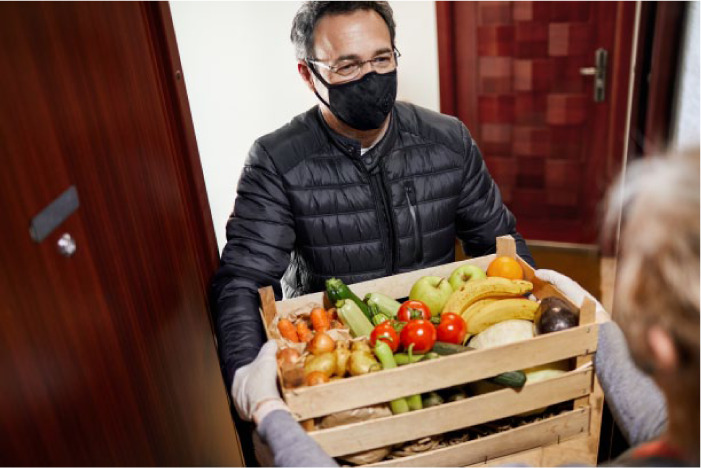


The scale of community action in the UK since the start of the COVID-19 pandemic has been significant. Community-based organisations, national charities, mutual aid groups and thousands of individual volunteers, including the 750,000 NHS volunteer responders, have stepped forward to support those made more vulnerable by the pandemic.^1,2^ These acts of community and solidarity between people range from practical help with shopping and running food banks, to telephone befriending and staffing helplines. In some areas, volunteers have even mobilised to conduct community-led contact-tracing to support the pandemic response.^3^

Community action is a vital part of the public health effort in a pandemic.^4,5^ Fundamentally, it is through the action of community members, via the majority of people adhering to social distancing, respiratory and hand hygiene recommendations, that the spread of a disease is attenuated.^6^ It is only through community support that those who are clinically vulnerable and who are required to ‘shield’ by staying at home for a protracted length of time can successfully self-isolate and maintain social distancing.^6^ Community mobilisation in an outbreak can also lead to growth of supportive community networks.^5^ The Office for National Statistics (ONS), who are tracking social impacts of COVID-19 in the UK, reported in May 2020 that just under 2 in 3 adults (64%) felt other local community members would support them if they needed help, and more than half (55%) had checked in with their neighbours.^7^

As the long-term social, health and economic impacts of COVID-19 are felt throughout the UK, communities and community-based organisations will have a critical role to play in the recovery process. The ability of communities to cope with and recover from large-scale emergencies is often referred to as ‘community resilience’.^8^ WHO Regional Office for Europe has long argued that building resilient communities and supportive environments is a public health priority.^9^ Creating resilient communities is not about preparing them to cope with shocks and their aftermath alone, rather it is about what public health systems can do to strengthen protective factors, such as strong social networks, which will aid people and communities to manage, adapt, and ultimately recover well.^10^ Indeed, literature demonstrates that being a member of multiple social networks or groups can have important effects for health and wellbeing, particularly during times of change.^11^

The UK public health community has a major part to play in strengthening community resilience in the context of COVID-19. We should initially ask two key questions: (1) How can local public health systems support the least advantaged communities to become more resilient?; (2) Who is at risk of being ‘left behind’?^4^ We know that the economic conditions that drive health inequalities undermine resilience^12^ and affect people’s ability and resources to cope with emergencies.^13^ Not everyone is able to contribute to or benefit equally from community action and the COVID-19 pandemic will have left some more isolated and vulnerable. Voluntary and community sector (VCS) organisations have a public health role here, particularly grassroots organisations that are in touch with groups and individuals who face the greatest risks. The public health workforce who are community based, such as community pharmacists, teachers and community development workers, also hold vital knowledge of local needs and assets and are trusted sources of advice for many individuals and families.^14^

**Figure 1:**
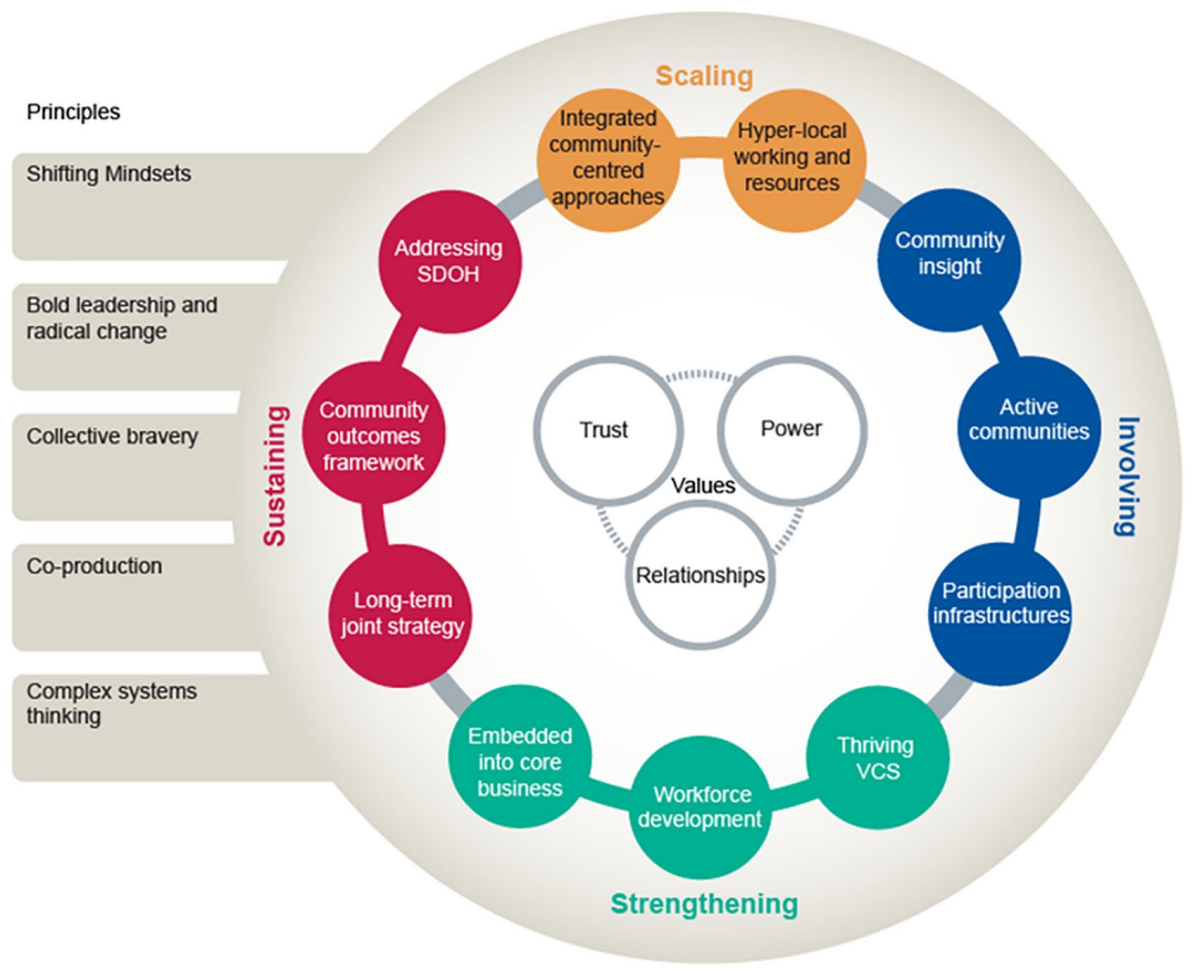
Eleven elements of community-centred public health: a whole system approach. Source: Public Health England.^16^ Figure 1 is made available under the Open Government Licence v3.0. (online): https://www.nationalarchives.gov.uk/doc/open-government-licence/version/3/.

Strengthening community resilience in the months and years to come will require a whole system approach working with different sectors. The UK Government Community Resilience Development Framework stresses the importance of enabling spontaneous community-led action alongside the need for public services to work in partnership with voluntary partners to coordinate efforts.^15^ Key routes for engagement include parish councils, local businesses, faith organisations, community hubs and resident associations. Public Health England (PHE) recently published guidance for taking a whole system approach to community-centred public health.^16^ This draws on international evidence and the experience of local authorities and public health leaders who are changing the way local public health is practised. While this guidance was developed before the pandemic, nonetheless the main elements, values and principles (Figure 1) can be used to guide a strategic response to strengthening community resilience in the recovery from the multiple impacts of the COVID-19 pandemic. We have highlighted how these might be applied in Table 1. A whole system approach to strengthening community resilience should also involve good public health intelligence. A WHO Europe report recommends assessing community vulnerabilities and capabilities across key economic and social indicators in combination with gathering insights from the most marginalised groups.^17^ Having a shared understanding of local assets and vulnerabilities will help in planning actions in the recovery period.

**Table 1:** Applying whole system community-centred public health to the COVID-19 recovery

Whole system community-centred public health^16^	Potential actions to build community resilience
Scaling	Take a place-based approach to reducing health inequalities, working across neighbourhoods. Starting at a ‘hyper-local’ level will tap into local community action and resources. Utilise community-centred approaches to provide support, alongside professional-led services. Local services such as social prescribing can offer a flexible, person-centred approach to supporting people during and after the emergency.
Involving	Maintain two-way communication and decision-making between communities and services, to ensure needs and priorities are understood and addressed. Establish new ways of gathering the insights of people most affected by the COVID-19 pandemic. Use community development methods, especially in marginalised communities, in order to increase people’s control over their health and wellbeing.
Strengthening	Work in partnership with local VCS organisations who are reaching out to those in need. Find ways to build the capacity of grassroots organisations that have close ties with marginalised and vulnerable groups. Support volunteering, working alongside volunteer-involving organisations to ensure volunteers have the right information, support and training to help people safely in the community. Enhance the skills of the public health workforce in community engagement and using asset and strengths-based approaches when tackling local priorities.
Sustaining	Prioritise meeting basic needs through action on employment, housing, food, income, debt, natural environment and education as building blocks for community resilience. Developing a strategic and long-term ambition for strengthening communities in the recovery from COVID-19. Improve measurement using community outcome frameworks with short-, medium- and long-term indicators on what matters to communities, such as a sense of belonging, or mental wellbeing.

Looking forward to the recovery from the COVID-19 pandemic, a collaborative approach to rebuilding community health, wealth and wellbeing will be needed; an approach that ensures that the health gap is reduced not widened. Many public health teams will be thinking now of how to build on the current levels of community action in planning for recovery. Many local authorities will have developed partnership arrangements with the voluntary and community sector that have withstood the test of this pandemic, having demonstrated capability to respond quickly to community needs. The potential to learn from these whole system approaches is huge, and work to capture lessons identified will be vital. In addition, those working and volunteering in communities through this outbreak will have gained important insights about what does and does not work when implementing initiatives ‘on the ground’.

In conclusion, we know that the economic, health and social impacts from the COVID-19 pandemic will be significant and unequally distributed. There is much that the public health community can do to work in solidarity and partnership with communities and community-based organisations to mitigate these impacts and to create the conditions for individuals and communities to thrive post-pandemic. This is an opportunity to create a new way of working and to realise the ambition of a more community-centred system building upon the community-driven initiatives that have been so important to the pandemic response to date.
